# The Odds of One-Year Mortality in Bedridden Geriatric Patients Discharged from Acute Rehabilitation Ward Are Increased Eightfold If the Patients Have Three or More Complications

**DOI:** 10.3390/jcm13020537

**Published:** 2024-01-17

**Authors:** Jure Aljinović, Blaž Barun, Ana Poljičanin, Darko Kero, Marija Matijaca, Dora Dujmović, Ivanka Marinović

**Affiliations:** 1 Institute of Physical Medicine and Rehabilitation with Rheumatology, University Hospital Split, Šoltanska 1, 21000 Split, Croatia; bbarun@kbsplit.hr (B.B.); ana.poljicanin@ozs.unist.hr (A.P.); mamatijaca@kbsplit.hr (M.M.); dodujmovic@kbsplit.hr (D.D.); imarinov@kbsplit.hr (I.M.); 2 University Department of Health Studies, University of Split, 21000 Split, Croatia; 3 Study Program of Dental Medicine, School of Medicine, University of Split, 21000 Split, Croatia; darko.kero@mefst.hr

**Keywords:** bedridden, geriatric, major illness, rehabilitation, mortality

## Abstract

Low muscle strength, functional score at discharge, and complications during a ten-day rehabilitation hospital stay can affect mortality rates in bedridden geriatric patients. This was a prospective observational study in a cohort of 105 bedridden geriatric patients admitted to the Rehabilitation ward after a major illness or surgery. All participants had a severe dependency on another person (Barthel’s Index < 60). The one-year mortality rate in this cohort was 15.2%, with further subdivision according to the number of complications: 61.5% in patients with ≥3 complications during hospitalization, 17.6% in patients with two complications, 9.5% with one complication, and 3% in patients with no complications. The Barthel Index at discharge (OR = 0.95; *p* = 0.003) and ≥3 medical complications (OR = 8.33; *p* = 0.005) during rehabilitation ward stay were significant predictors for one-year mortality. The odds of one-year mortality after discharge increased eightfold in patients with ≥3 medical complications. Sarcopenia, age, and sex were not significant predictors of mortality in this cohort. The 10-day acute rehabilitation was too short to achieve progress from severe to moderate independence in 60% of patients. The Barthel Index at discharge and a number of complications affect the mortality rate. These findings provide valuable insights into the complex dynamics of mortality and functional outcomes in bedridden geriatric patients.

## 1. Introduction

The World Health Organization (WHO) introduced the “Decade of Healthy Ageing” from 2020 to 2030 with the start of older age chronologically set at ≥65 and adopted by some insurance companies (Medicare in the USA). Geriatrics refers to medical care for older adults who may have a decline in physiological reserve in organs, polypharmacy, and increased complexity of medical comorbidities. Sometimes individuals as young as 55 who require nursing-home-level care may be considered geriatric patients [1].

Falls are the leading reason for hospitalization in geriatric patients. The omission of physical therapy during a hospital stay can lead to increased dependency on others for assistance. Studies have shown that approximately one-half of geriatric patients report decreased ability to perform daily activities and 65% report a lower level of mobility after extended hospitalization [2,3].

Geriatric patients can even become bedridden after a major illness or surgery. These patients often require a level of care provided in nursing homes, which can be a burden for both their families and society. In some countries, they may not have access to physical therapy as ambulatory patients do.

The reason for the progressive loss of function after a major illness or injury is a combination of catabolic processes in the body, accelerated muscle loss, and the decline in skeletal muscle protein synthesis [4]. The postoperative decline in muscle function was first documented by Edwards et al. [5]. Muscle loss can lead to sarcopenia, which is associated with a higher mortality risk in the elderly (the pooled hazard ratio of sarcopenia is 1.21) [6].

Resistance exercises and nutritional support are beneficial in slowing down muscle atrophy [7,8]. Exercises are crucial as they provide a strong anabolic stimulus. Therefore, early physical therapy (sitting, upright standing, walking with crutches or walker, and resistance exercises) along with long-term physical medicine planning is imperative for geriatric patients to regain ambulatory function after major illness or surgery.

Remaining bedridden after physical therapy was pointed out as a predictive factor of mortality [9]. A low functional index like the Barthel index was associated with higher mortality in elderly patients after hip fracture [10] and after stroke [11].

At our acute stationary rehabilitation department at Clinical Hospital Split, we provide ten-day physical therapy for bedridden geriatric patients who are physically capable of exercising but have significant comorbidities preventing them from being discharged for home rehabilitation or to chronic rehabilitation institutions.

The objective of this study was to follow up a cohort of 105 geriatric bedridden patients for one year and to determine whether sarcopenia at admission, functional score at discharge, or medical complications during hospital stay affect the mortality rate.

## 2. Methods

### 2.1. Study Design

This prospective observational study was conducted from July 2021 to September 2022 at the Institute of Physical Medicine and Rehabilitation with Rheumatology, University Hospital Split in Croatia. The study was conducted in accordance with the Declaration of Helsinki and approved by the Ethical Committee of the University Hospital Split (500-03/20-01/86, 24 August 2020.).

This study is part of the University of Split’s project, registered as OZS-IP-2020-1: “Improvement of Access to Physical Therapy for Immobile Geriatric Patients at the Institute for Physical Medicine and Rehabilitation with Rheumatology”.

All participants were informed about the study design and informed consent was obtained.

The study was reported according to Strengthening the Reporting of Observational Studies in Epidemiology (STROBE).

### 2.2. Participants

Enrolled subjects met the following inclusion criteria:

Patients who were older than 65 (defined as geriatric patients only by chronological viewpoint) who were bedridden just recently, by serious illness, and admitted to the Institute of Physical Medicine and Rehabilitation at University Hospital Split with severe dependency on another person as measured by Barthel’s Index <60.

Patients who lacked the capacity to provide informed consent or who declined to participate in the study were excluded.

The final number of enrolled participants was 105. An additional 15 participants declined to sign the written consent and were not included in the study. Patient identity was coded with the unique patient number known only to the principal investigator.

### 2.3. Data Measurement

Baseline demographics, anthropometric data, and the condition that led to hospitalization were noted.

Questionnaires were administered at admission and upon the patient’s discharge.

The Barthel Index (also known as the Barthel Score) is a widely used assessment tool to measure an individual’s functional independence in activities of daily living (ADL). It was assessed twice, upon admission and at discharge (10 days apart). During the ten-day hospital stay, patients underwent passive and active exercises for major muscle groups, respiratory exercises, verticalization, and assistance with standing or walking twice a day with the use of a walker or crutches. The Barthel index was administered by experienced physiotherapists as a part of routine geriatric assessment at our Clinic without physiotherapists knowing about the study.

The Barthel Index was developed by Mahoney and Barthel in 1965 and has since become a standard measure in healthcare settings, particularly in rehabilitation and geriatric care. The Barthel Index consists of ten ADL items, including feeding, bathing, grooming, dressing, toileting, bladder and bowel control, transferring (e.g., moving from bed to chair), mobility (e.g., walking or using a wheelchair), ascending and descending stairs, and dressing. Each item is scored based on the person’s ability to perform the task independently, with combined scores of all ten items ranging from 0 to 100. The results are interpreted according to dependency on the help of another person: 0–20: total dependency; 21–60: severe dependency; 61–90: moderate dependency; 91–99: slight dependency.

The SARC-F questionnaire is a tool designed to assess the risk of sarcopenia and is regarded as a screening method. SARC-F was compared to hand-held dynamometry testing of grip strength, which was validated as a muscle strength method. It is also regarded as a method for probable diagnosis of sarcopenia. Grip strength moderately correlates with strength in other body areas, making it a reliable surrogate for more complicated measures of arm and leg strength, and is recommended for routine use in hospital practice [12]. Dynamometry testing is expressed in kilograms and a validated instrument was used for the measurements (Jamar Hydraulic Handheld Dynamometer, Sammons Preston, Inc., 119 Bollingbrook, IL, USA).

Participants measured grip strength three times with each hand, and the highest recorded value was used for calculation. Probable diagnosis of sarcopenia was defined as hand grip strength of less than 27 kg for males and less than 16 kg for females as defined by the European Working Group on Sarcopenia in Older People (EWGSOP) [12].

Physiatrists responsible for the patients recorded all medical complications that occurred during the ten-day hospitalization in the Rehabilitation department. One-year mortality was analyzed using obligatory health insurance registers, where the date of death is recorded.

## 3. Outcomes

The primary outcome of this study was to analyze the effect of low muscle strength, functional score at discharge, and a number of complications during Rehabilitation ward stay on one-year mortality in geriatric bedridden patients.

Secondary outcome measures included changes in the functional index at admission and discharge measured by Barthel’s index, establishing the incidence of probable sarcopenia in bedridden geriatric patients measured by hand grip dynamometry testing, and establishing the prevalence of medical complications during their stay at the Rehabilitation ward.

## 4. Statistical Analysis

Continuous data, such as age, Barthel score, and dynamometry results, were presented as means with standard deviations (SD) and 95% confidence intervals (CI). Differences in Barthel scores and dynamometry measurements between groups were assessed using a *t*-test and linear regression after coding groups as dummy variables.

To model the probability and odds of one-year mortality, we used logistic regression models with the one-year mortality as the binary outcome variable and the Barthel Index, dynamometry, presence and number of complications during rehabilitation ward stay, age, and gender as predictors. Several logistic regression models were created and the relative quality of each model was estimated using Akaike’s Information Criterion (AIC).

Statistical analysis was entirely performed in Microsoft Office Excel 2016 32–bit (Microsoft Corporation, Redmond, WA, USA). The level of statistical significance was set at α = 0.05 (*p* < 0.05).

## 5. Results

Between July 2021 and October 2022, a total of 105 bedridden geriatric participants were enrolled in the study. This cohort consisted of 70.5% (74/105) females and 29.5% (31/105) males. Participants were divided into three groups for additional analyses: young-old (65 to 74), middle-old (75 to 84), and oldest-old (85+). Notably, there was a consistent increase in the female-to-male ratio across these groups, with ratios of 1.69, 2.7, and 3.2 observed within the respective groups (Table 1).

The most common reason for hospitalization was proximal femur fracture surgery, accounting for 56 cases. This included intramedullary pin osteosynthesis in 33 participants, biarticular hip endoprosthesis in 11, partial hip endoprosthesis in 10, and total hip endoprosthesis in 2 cases. Neurological diseases were the second most common cause of hospitalization, observed in 21 participants, with paraparesis in 10, hemiparesis in 9, and subdural hematoma in 2 cases. Fractures in locations other than the femoral bone were found in 16 cases, followed by lower limb amputation in 7 cases and degenerative osteoarthritis in 5 cases. The breakdown of reasons leading to immobility in geriatric patients and subsequent referrals to the Rehabilitation ward is summarized in Table 2.

A handheld dynamometer was used to measure grip strength. Participants measured their grip strength three times with each hand, and the highest recorded value was used for calculation. The results showed no significant difference in grip strength between the right and left hands for both genders (19 ± 9.9 vs. 17.86 ± 9.73 kg, right vs. left, *p* = 0.244). Females had lower grip strength than males (19.15 ± 7.4 vs. 26.8 ± 11.5 kg, *p* = 0.0001).

A probable diagnosis of sarcopenia was defined as hand grip strength of less than 27 kg for males and less than 16 kg for females. Sarcopenia was found in 32.35% of bedridden patients (*n* = 33 out of 102), while 67.65% (*n* = 69 out of 102) did not have sarcopenia. Males had sarcopenia in 51.56% (*n* = 16 out of 31), while females had sarcopenia in 23% of cases (*n*= 17 out of 71). Linear regression analysis indicated that there was no significant difference in grip strength based on age groups (*p* = 0.431).

A comparison between the SARC-F questionnaire (a sarcopenia screening method) and hand dynamometry testing was conducted. SARC-F yielded accurate results in 48% of the geriatric bedridden patients (49/102), while it produced false-positive results in 50% of cases (51/102). In only 2% of cases (*n* = 2 out of 102), there was a false-negative result. The specificity of SARC-F was 29%, the sensitivity was 93%, the positive predictive value was 38.27%, and the negative predictive value was 90.9%.

During the ten-day stay, 72 participants (69%) experienced complications that required medical intervention. Most participants had one complication (42 patients), while 17 patients developed two complications, and 13 patients had three or more complications. Urinary tract infections were diagnosed in 51 cases, followed by 11 cases of pressure ulcers, 9 patients with delirium, 8 cases of respiratory tract infections, and 5 patients requiring blood transfusions due to anemia.

Thirty-three participants (31%) had no complications. All complications and their incidence can be found in Table 3.

Barthel’s score was analyzed in all patients and then categorized into different groups:

For all patients: Barthel’s score at admission to the Department of Rehabilitation Medicine (Barthel 1) was 32.82 ± 15.04, while the discharge score (Barthel 2) was 51.4 ± 23.38 (*n* = 102).

According to gender: In both genders, Barthel’s scores increased from admission to discharge after ten days of hospital physical therapy. For males, it was 29 ± 13.27 vs. 45.45 ± 20.7 (Barthel 1 vs. Barthel 2; *n* = 29), while in females, it was 34.34 ± 15.51 vs. 53.77 ± 24.09 (*n* = 73). All of these differences were statistically significant (*t*-test, *p* < 0.001), with average means of Barthel score higher in females.

According to the occurrence of complications: Participants who did not experience complications during their stay in the Rehabilitation ward (Ncomp = 0) had a Barthel 1 score at admission 38.59 ± 13.82, and the discharge score was 59.86 ± 19.38 (*p* < 0.0001, *t*-test). Participants who had one or more complications during their ten-day stay (Ncomp ≥ 1) had a Barthel 1 score of 30.53 ± 14.98 and a Barthel 2 score of 48.04 ± 24.1 (*p* < 0.0001, *t*-test). Although both groups showed an improvement in the functional index after physical therapy, linear regression analysis revealed a statistical difference in the discharge score (Barthel 2) between the groups with and without complications, with the average mean score being higher in the group without complications (Barthel 2, *p* = 0.021). The most significant difference was found in the Barthel 2 score in the group of patients with three or more complications (Ncomp ≥ 3), which was 28.67 lower than in all other groups combined (26.38 ± 21.47 vs. 55.06 ± 21.42).

The analysis of one-year survival rates in bedridden geriatric patients after their discharge from the rehabilitation ward revealed that 84.56% (89 out of 105) were alive after one year. The one-year mortality rate was 15.24%, with 16 out of 105 participants passing away within the first year. Among the deceased patients, the average age was 81.5 ± 5.2 years.

On average, participants who survived one year after the discharge had a Barthel 2 score 28.78 points higher than the deceased participants (*p* < 0.0001).

Patients who experienced three or more complications during their stay had a mortality rate of 61.5% (8 out of 13), those with two complications had a mortality rate of 17.6% (3 out of 17), those with one complication had a mortality rate of 9.5% (4 out of 42), and patients with no complications had a mortality rate of 3% (1 out of 33). Ten patients passed away in the first trimester after discharge, followed by one in the second trimester, one in the third trimester, and four in the fourth trimester.

There was no statistically significant difference in hand dynamometry scores between patients who survived one year and the deceased ones (*p* = 0.058). Additionally, there was no statistically significant difference in hand dynamometry scores between the groups with or without complications during their stay in the Rehabilitation ward.

**Figure 1:**
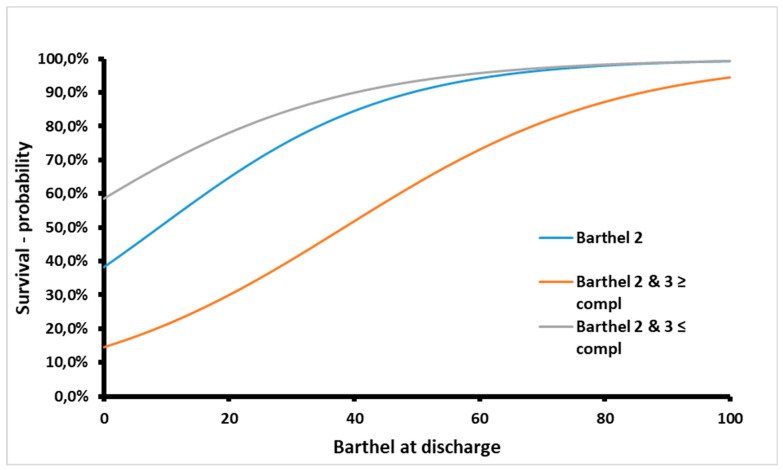
The optimal predictive model for estimating the one-year survival probability of geriatric bedridden patients following their discharge from a ten-day acute stationary rehabilitation program is based on two key factors: the Barthel Index score at the time of discharge (Barthel 2) and the number of complications experienced during the rehabilitation period.

**Figure 2:**
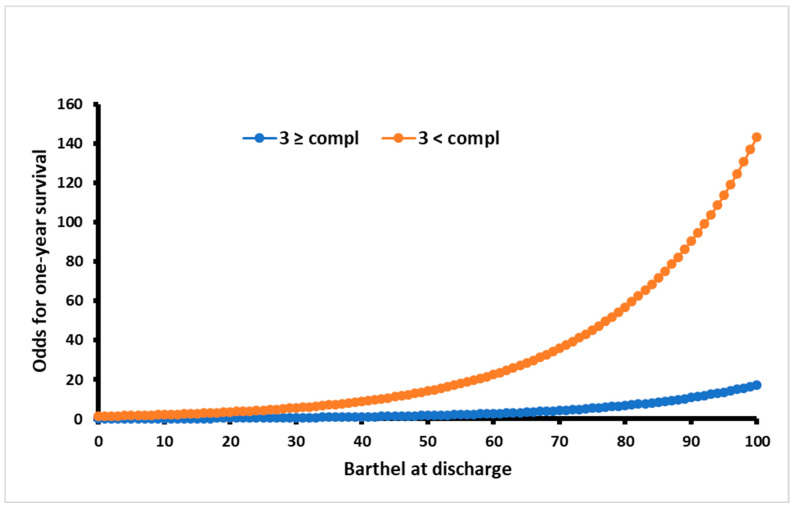
Odds for survival probability in patients with ≥3 or <3 medical complications in respect to discharge Barthel.

Barthel Index at discharge (OR = 0.95; *p* = 0.003) and ≥3 medical complications (OR = 8.33; *p* = 0.005) during rehabilitation ward stay were significant predictors for one-year mortality of geriatric bedridden patients after severe illness or major surgery (Table 4.). The most accurate model for predicting one-year survival was based on these two predictors (Figure 1). This model showed an accuracy of 89% (AIC 62.89, *p* < 0.0001, α = 0.05, logistical regression method). The rest of the models are presented in Table 4. The odds for the first-year survival probability were eight times lower for patients with three or more medical complications (Figure 2).

## 6. Discussion

Our cohort of geriatric bedridden patients, who required rehabilitation following major illness or surgery, exhibited a one-year mortality rate of 15.2%. Among these patients, half underwent hip fracture surgery (50%) and an additional 10% underwent surgery for middle or distal femoral bone fractures. Our cohort’s mortality rate falls within the lower range of rates reported in the literature, ranging from 14% to 58% after hip surgery, even smaller than the latest 1-year mortality of 23.3% reported in Europe by Downey et al. in 2019 [9,13]. It is worth noting that these numbers may not be directly comparable due to the high heterogeneity in the diagnoses of our patients. The results directly comparable to ours were published by Xu et al. who detected a similar one-year mortality of 16% in the geriatric rehabilitation inpatient population [14].

The analysis of mortality predictors was conducted to identify independent and modifiable factors that could impact future interventions.

Advanced age, both after hip fracture and stroke, was observed to be an independent predictor of one-year mortality in the literature [15,16,17]. Also, age and cognitive level were already independently related to the functional level at discharge [18]. Although we observed the older age of the deceased patients in this study (81 ± 5 vs. 77 years), age was found not to be a statistically significant variable for the prediction of mortality.

Modifiable variables such as functional index at discharge, complications during the rehabilitation process, and sarcopenia existence, were discussed separately.

(a) Functional index: Our acute 10-day rehabilitation was found to be insufficient for transitioning patients from severe to moderate dependency, as approximately 60% of patients remained below a Barthel Index score of 60 (only 42 patients were above BI 60). Remaining bedridden after physical therapy was pointed out as a predictive factor of mortality [9]. A low postoperative Barthel index was associated with higher mortality in elderly patients after hip fracture [10]. A low Barthel index was also found to be a predictor of mortality after stroke [11]. This is in accordance with the finding of this study that 87.5% of deceased patients remain bedridden after discharge with a Barthel index of less than 60, and a significantly lower Barthel index than other patients. Malafarina et al. addressed the same problem and proposed a 40-day rehabilitation program after hip surgery, which led to a Barthel Index score above 60 for the majority of patients [19].

(b) Medical complications during the rehabilitation process like urinary tract infection, pulmonary infection, and pressure ulcers are known to affect functional score at discharge and increase mortality [16,20]. Hospital-treated infections are rising according to the literature [21]. Pressure ulcers as a complication increased mortality risk in hospitalized older patients by two times [18,22]. Intensified geriatric care was used in patients with a risk for developing pressure injuries: pressure-relieving mattress, heel-elevating cushions, neutral skincare products, and changing positions in bed.

In this study, the prevalence of all pressure sores was 10.4% which is in accordance with meta-analysis data of pressure injury prevalence of any stage in geriatric patients of 11.6% [23]. Only two persons with pressure sores died in the first year so it is not useful as o variable for prediction of mortality in this study.

Prevention of hospital falls by using bed rails in at-risk patients and short-term usage of physical restraint (in patients with postoperative delirium after psychiatrist recommendation) helped us limit the fall rate to 2%. This is on the lower end of the reported fall rate of 2–17 falls in 1000 patient days [24]. Postsurgical delirium is yet another factor associated with greater morbidity and mortality and was identified as a predictor of poor prognosis in hip fracture patients [25]. The prevalence of delirium in geriatric patients in different hospital wards ranged from 11–42%, and in this paper, we observed a prevalence of 9%, which is on the lower end of the spectrum [26].

The combined incidence of urinary or respiratory infections, pressure ulcers, postoperative anemia, and phlebothrombosis occurred in more than half of our bedridden geriatric patients. The number of complications during this study was similar to the paper from Poh et al. in 2013 where 56% of all patients had at least one complication, with urinary tract infection or retention as the most common diagnosis [27]. In this study, patients with three or more complications during rehabilitation had a 61% mortality rate, which is a drastic increase from 17% in patients with two complications and even more than 3% in patients with no complications. The high mortality rate of patients with three complications can only be compared to critically ill patients who are admitted to intensive care units and have a one-year mortality rate of 33–72% [28] and critically ill patients admitted to emergency departments with mortality rates up to 88% [29].

Addressing medical conditions that may interfere with physical therapy treatment is crucial for the success of rehabilitation, which can be measured by a better functional index at discharge.

(c) Sarcopenia increases mortality in elderly people [6]. The prevalence of sarcopenia in this study cohort was 32.35%, which is similar to the prevalence reported in nursing homes by Senior et al. (32%) [30] but higher than in the general elderly population (10–16%) [31]. There was a higher incidence of sarcopenia in males compared to females among bedridden geriatric patients (51% vs. 23%), consistent with findings in studies involving hip fracture [32] and stroke patients [33]. Sarcopenia was not a contributing factor in the mortality prediction model in this study, unlike the studies where the increase in mortality was found in the osteosarcopenia group of patients after hip fracture [34] and bedridden patients with sarcopenia [14]. According to other studies, sarcopenia presence was associated with having lower mobility at one year [35], and sarcopenia treatment was connected to a positive, but not statistically significant, effect on the recovery of daily living in the geriatric population after hip fracture [19]. Low muscle strength was not connected to increased one-year mortality [14].

The presence of sarcopenia can significantly limit the success of rehabilitation due to low muscle strength and function. According to the EWGSOP, low muscle strength is identified as the key feature of sarcopenia and sufficient to start the treatment [12]. However, in hospital settings, particularly among bedridden geriatric patients, definitively diagnosing sarcopenia can be challenging because procedures such as DXA, MR, and CT scans, which assess muscle quality and quantity, are often impractical and inaccessible, primarily remaining within the domain of scientific research.

Bioelectrical impedance analysis could be a promising method for analyzing muscle mass in geriatric patients, but many of the patients in this study, having suffered hip fractures or hemiplegia following a stroke, were unable to maintain an upright position for the necessary measurements. This limitation also applies to physical performance tests that assess muscle functionality and the severity of sarcopenia, such as the Chair Stand test, Timed-Up-and-Go test, Gait Speed test, 400-m walk, or long-distance corridor walk test.

Croatian legislation follows the EWGSOP recommendations, and either low grip strength or SARC-F result of ≥4 is enough for the prescription of oral nutritional supplementation.

In our study of geriatric bedridden patients, we observed a notable inconsistency when comparing SARC-F results with grip strength. In contrast to previous validation studies, which indicated low to moderate sensitivity (28.9% to 55.3%) and high specificity (68.9% to 88.9%) of SARC-F in detecting sarcopenia [36], our results showed high sensitivity (93%) and low specificity (29%) of SARC-F when correlated with low grip strength in this specific population. This was the result of a high number of false positive results (50%). All bedridden patients cannot climb stairs and cannot rise from a chair and then automatically have a SARC-F score of 4, so this sudden immobile state of the patient was diametrically different a day before the fall in most of the patients. Therefore, the modified version of the SARC-F test should be developed to increase the specificity of the SARC-F test for geriatric bedridden patients in hospital settings. We propose modifying questions by adding “7 days before the reason (stroke, fall) for bedridden state occurred” to clarify the patient’s condition before the major illness or fall occurred (e.g., Could you climb the stairs 7 days before the fall?).

Instead of SARC-F, hand dynamometry might be recommended as a routine procedure in hospital settings for diagnosing sarcopenia. This procedure is easy to perform for patients and is repeatable. According to our findings, there was no difference in hand grip strength between dominant and non-dominant hands in both genders so it can be safely used in hemiplegic patients. Hand grip strength was not affected by age since it was not statistically different in young-old, middle-old, and oldest-old groups. Gender was the only relevant factor for hand grip strength (lower in women), and it confirmed EWGSOP cut-off values. Interestingly, our retrospective analysis found no initial differences in hand grip strength between deceased and surviving patients.

## 7. Conclusions

To conclude, the Barthel Index at discharge and three or more complications were significant predictors for one-year mortality of geriatric bedridden patients during rehabilitation ward stays after severe illness or major surgery. The odds of first-year mortality after discharge from the Rehabilitation ward increased eightfold in geriatric bedridden patients with three or more complications.

Other conclusions are presented point by point:

Sarcopenia occurrence in geriatric bedridden patients defined with low muscle strength was 32% (more prevalent in males 51% than in females 23%).

There was no difference between dominant and non-dominant hand grip strength in geriatric bedridden patients.

Age did not affect hand grip strength.

Female gender was associated with lower grip strength (19.15 ± 7.4 vs. 26.8 ± 11.5 kg).

The first-year mortality rate in this cohort was 15.2%, with further subdivision according to the number of complications: 61.5% in patients with three or more complications during a hospital stay, 17.6% in patients with two complications, 9.5% with one complication, and 3% in patients with no complications.

The presence of sarcopenia did not affect the mortality rate (*p* = 0.058).

Acute 10-day rehabilitation was proven to be too short for progressing from severe to moderate independence since around 60% of the patients remained below the Barthel Index score of 60.

Future studies should investigate how the interventions for sarcopenia, osteoporosis, and prevention of medical complications affect the mortality rate in geriatric bedridden patients. Geriatric patients with a lower functional index and three or more complications require more frequent medical controls and a longer duration of stationary type of rehabilitation. The findings from this study provide valuable insights into the complex dynamics of mortality and functional outcomes in geriatric bedridden patients, offering guidance for future interventions and care strategies in this vulnerable population.

## 8. Limitations of the Study

The study has some limitations that need to be considered. Firstly, due to the high heterogeneity of the diagnoses, it was not possible to analyze patients with less common diagnoses. Secondly, since 14% of the patients refused to participate in the study, there is a possibility of selection bias. To overcome these limitations, future studies should include more patients with different diagnoses. Additionally, this study did not include comorbidity index scores, which should be considered in future studies. Lastly, low muscle strength was used as a surrogate measure for the diagnosis of sarcopenia, but a definitive diagnosis could not be established in bedridden hospital patients.

## Figures and Tables

**Table 1:** Subdivision of geriatric bedridden patients by age.

Age	*n*	%	f	m	Ratio f/m
65–74	35	33.33%	22	13	1.69
75–84	49	46.67%	36	13	2.77
85+	21	20.00%	16	5	3.20
Total	105	100.00%	74	31	2.39

The average age of the participants was 77 ± 6.67 years (mean ± standard deviation), with no statistically significant difference observed between genders (78.54 ± 6.47 for females vs. 76.65 ± 7.07 for males, *p* = 0.186, *n* = 105).

**Table 2:** The reason for hospitalization and development of immobility.

Reason for Referral to Rehabilitation Ward		Total *n*
Proximal femoral fractures		56
	Intramedullary pin osteosynthesis 33	
	Biarticular hip prosthesis 11	
	Partial hip endoprosthesis 10	
	Total hip endoprosthesis 2	
Fractures of other localization		16
	Acetabular fracture 3	
	Vertebral fracture 5	
	Car accident multiple fractures 5	
	Periprosthetic knee fracture 3	
Osteoarthritis		5
	Total endoprosthesis of the knee 3	
	Total endoprosthesis of the hip 2	
Lower limb amputation		7
	Transfemoral amputation 5	
	Transtibial amputation 2	
Neurological diseases		21
	Hemiparesis due to stroke 9	
	Paraparesis due to thoracic spinal cord meningioma (operation) 3	
	Guillain-Barré syndrome paraparesis 3	
	Post-COVID paraparesis 3	
	Spondylodiscitis 1	
	Head trauma and bleeding 2	

**Table 3:** Complications that required medical assistance during the hospitalization.

Complication:		Total *n*
Uroinfection		51
Respiratory tract infection group		8
	pneumonia 3 cases	
	tonsillopharyngitis 4 cases	
	COVID-19 1 case	
Pressure ulcers		11
Delirium		9
Gastrointestinal condition		7
	Cholecystitis acuta 2 cases	
	Paralytic ileus 1 case	
	Nasogastric tube placement 3 cases	
	Haematochezia 1 case	
Infective diarrhea		5
	Clostridium difficile 3 case	
	Unknown pathogen 2 cases	
Cardiovascular condition		15
	Phlebothrombosis in legs 3 cases	
	Pulmonary embolism 1 case	
	Symptomatic anemia 5 cases	
	Myocardial infarction 1 case	
	Warfarin intoxication 1 case	
	Paroxysmal atrial fibrillation- 2 cases	
	Unregulated hypertension- 1 case	
	Cardiac decompensation-1 case	
Falls		2
Presyncope of unknown cause		4
Other		11

**Table 4:** Statistical parameters for different models used to determine one-year survival probability based on one or more variables.

						95% CI			
	df	Predictor(s)	Coeff.	*p*-Value Coeff.	Odds Ratio	Lower	Upper	AIC	ΔAIC
MODEL 3	2	Barthel 2	0.046	0.003	1.047	1.015	1.08	62.896	0
		3 ≥ compl	−2.129	0.005	0.12	0.027	0.536		
MODEL 1	3	Dynamo.	0.022	0.53	1.02	0.955	1.093	64.731	1.835
		Barthel 2	0.047	0.003	1.048	1.016	1.081		
		3 ≥ compl	−19.22	0.998	4.51996 × 10^−9^	0	N/A		
MODEL 2	1	Barthel 2	0.015	0.0001	1.056	1.026	1.087	67.633	4.737
MODEL 4	1	3 ≥ compl	−2.821	3.30577 × 10^−5^	0.059	0.016	0.226	75.684	12.78
MODEL 5	1	Dynamo.	0.06	0.06	1.062	0.997	1.131	85.31	22.415
MODEL 7	1	1 ≥ compl	−2.031	0.054	0.131	0.017	1.04	87.446	24.549
MODEL 6	1	Age	−0.104	0.023	0.901	0.823	0.986	87.904	25.008

Legend: Barthel 2—Discharge Barthel Index, Dynamo—Hand grip dynamometry, AIC—Akaike Information Criterion (AIC), an estimator of prediction error.

## Data Availability

Anonymous data can be provided upon request.
